# Advanced central nervous system imaging biomarkers in radiologically isolated syndrome: a mini review

**DOI:** 10.3389/fneur.2023.1172807

**Published:** 2023-05-19

**Authors:** Sara Collorone, Michael A. Foster, Ahmed T. Toosy

**Affiliations:** Queen Square MS Centre, Department of Neuroinflammation, UCL Queen Square Institute of Neurology, Faculty of Brain Sciences, University College London, London, United Kingdom

**Keywords:** multiple sclerosis, magnetic resonance imaging, optical coherence tomography, connectivity, neurodegeneration, radiologically isolated syndrome

## Abstract

Radiologically isolated syndrome is characterised by central nervous system white-matter hyperintensities highly suggestive of multiple sclerosis in individuals without a neurological history of clinical demyelinating episodes. It probably represents the pre-symptomatic phase of clinical multiple sclerosis but is poorly understood. This mini review summarises our current knowledge regarding advanced imaging techniques in radiologically isolated syndrome that provide insights into its pathobiology and prognosis. The imaging covered will include magnetic resonance imaging-derived markers of central nervous system volumetrics, connectivity, and the central vein sign, alongside optical coherence tomography-related metrics.

## 1. Introduction

The increasing use of magnetic resonance imaging (MRI) scans in clinical practice has led to the finding of central nervous system (CNS) abnormalities suggestive of multiple sclerosis (MS) in the absence of clinical episodes. In 2009, Okuda et al. (1) characterised this phenomenon by identifying the radiologically isolated syndrome (RIS), a condition linked to the future development of MS. Risk factors for conversion include younger age at diagnosis, the presence of spinal cord lesions, dissemination in time (DIT), and the presence of oligoclonal bands in cerebrospinal fluid (CSF) (1–3). The last two factors are included in the diagnostic criteria for MS if clinical symptoms are present (4). Whether RIS constitutes a prodromal phase of MS is still unclear—one study suggested that the 10-year risk of conversion to MS is just over 50% (3). RIS itself could be a subclinical form of MS which, although there is CNS damage, is still asymptomatic. Indeed, recent research has demonstrated that the use of dimethyl fumarate, a first-line disease-modifying treatment for multiple sclerosis, can delay the onset of MS in people with RIS (5).

This mini review presents the results of recent imaging developments applied to RIS to investigate CNS damage and highlights future challenges in the field. Literature search was performed on Embase, seeking articles matching both “Radiologically Isolated Syndrome” and either “Magnetic Resonance Imaging” or “Optical Coherence Tomography.” Relevant articles were selected after reviewing the title and abstract of each result, looking for those pertaining to volumetrics, quantitative MRI, the central vein sign, connectivity, or retinal layer imaging.

## 2. Advanced imaging biomarkers in RIS

### 2.1. Brain MRI

Ever since the definition of RIS in 2009 by Okuda et al. (1), researchers have tried to characterise and anticipate its clinical evolution. As MRI began to play a crucial role in predicting MS disease activity and progression from its onset (6, 7), many researchers investigated which MRI hallmarks distinguish individuals with RIS from healthy controls and people with MS. Whilst early research focussed on lesion characteristics (2, 8), later studies applied quantitative multi-modal MRI techniques to study normal-appearing tissues. The methods used included magnetisation transfer (MT), diffusion-weighted imaging (DWI), magnetic resonance spectroscopic imaging (^1^H-MRSI), and brain and spinal cord volumetrics.

An early study by De Stefano et al. (9) at 1.5T MRI reported that people with RIS and relapsing-remitting MS (RRMS) had similar global brain and cortical volumes, both lower than healthy controls. However, people with RRMS had a lower MT ratio (i.e., an index reflecting damage to the myelinated fibres) (10) in the normal-appearing brain tissue compared with RIS and healthy control participants. The authors concluded that this index of magnetisation exchange between brain macromolecules and water could be used to stratify the risk of MS conversion among RIS patients. However, their study had a cross-sectional design, with findings not yet confirmed in a longitudinal setting.

The same group further explored alterations in the normal-appearing brain tissue of individuals with RIS using ^1^H-MRSI at 1.5 T (11). N-acetyl aspartate (NAA) and choline (Cho) normalised to creatine (Cr) were measured in lesional/perilesional, normal-appearing white matter (WM) regions and the cortical grey matter (GM). They found lower NAA/Cr levels in RIS than in controls. NAA is synthesised in neurons and is considered a marker of neuronal integrity in the central nervous system (12). Its alterations have been widely reported in MS (13). Stromillo et al. (11) stratified RIS subjects according to the risk of developing MS based on established prognostic factors: spinal cord lesions, unmatched oligoclonal bands, and DIT. However, they could not find any differences in patients with different risk levels.

Another study (14) at 1.5 T confirmed the volumetric findings from De Stefano et al. (9). However, whilst the latter had included RRMS patients with a disease duration of up to 7 years, Rojas et al. (14) compared RIS subjects with people with clinically isolated syndrome (CIS) within 2 months of onset. They found similar global brain and cortical volumes for RIS and CIS, both lower than healthy controls.

The studies mentioned above did not investigate deep grey matter. However, Azevedo et al. (15), using 3T MRI (see Table 1), found a weak effect towards lower total GM volume in RIS compared with healthy controls. However, deep GM volume was significantly lower in RIS than in healthy controls, especially the thalamus. The authors also reported thinner right superior and inferior parietal cortical gyri in RIS than in controls.

**Table 1:** Summary of studies into advanced imaging biomarkers in radiologically isolated syndrome.

**Study**	**Design**	**Population**	**MRI scanner**	**Imaging protocol**	**MRI post-processing**	**Results**	**RIS stratification criteria**
**Brain volumetrics**							
De Stefano et al. (9)	Cross-sectional	• 19 RIS • 20 RRMS • 20 HCs	1.5 T	T1 gradient echo	FSL—SIENAX	• **Brain and cortical volume** RIS = RRMS < HCs • **NAWM volume** RIS = HCs > RRMS	NA
Rojas et al. (14)	Cross-sectional	• 10 RIS • 42 CIS (2 months from onset) • 29 HCs	1.5 T	T1 spin echo	FSL—SIENAX	• **Brain and cortical volume** • RIS = CIS < HCs • **NAWM volume** • RIS = CIS = HCs	NA
Azevedo et al. (16)	Cross-sectional	• 21 RIS • 42 HCs	3 T	3D T1	FreeSurfer (v5.2; Desikan-Killiany atlas)	• **Deep GM and thalamic volume** • RIS < HCs • **Brain, cortical and NAWM volume** • RIS = HCs • **R superior and inferior parietal gyri** • RIS < HCs	NA
Labiano-Fontcuberta et al. (17)	Cross-sectional	• 17 RIS • 17 CIS (12 months from onset) • 17 HCs	3 T	3D T1	FSL—SIENAX FreeSurfer (Talairach atlas)	• **Cortical and thalamic volume** • RIS < CIS = HCs • **Brain and NAWM volume** • RIS = CIS = HCs • **Cortical thickness** • RIS = CIS = HCs	No differences between groups^a^
Vural et al. (18)	Longitudinal (5 years median FU)	• 15 RIS • 15 HCs	3 T	3D T1	SPM12	• **Brain and thalamic volume** • RIS < CIS = HCs • **GM and NAWM volume** • RIS = HCs	NA
George et al. (19)	Cross-sectional	• 21 RIS • 38 HCs	3 T	3D T1	SPM12	• **GM (total, cortical and deep), NAWM and brainstem** • RIS = HCs • **Cerebellar WM and anterior GM** • RIS < HCs	NA
**Quantitative MRI**							
De Stefano et al. (9)	Cross-sectional	• 19 RIS • 20 RRMS • 20 HCs	1.5 T	MT	• MTr • brain, cortex, lesional and NAWM • Voxel-based analysis	• **Brain, NAWM and cortical MTr** • RIS = HCs > RRMS	NA
Stromillo et al. (11)	Cross-sectional	• 23 RIS • 20 HCs	1.5 T	^1^H-MRSI	• VOI corpus callosum (lesional and NAWM)occipito-parietal cortex • NAA/Cr • Cho/Cr	• **NAA/Cr levels** • RIS < HCs	No differences between groups^b^
Labiano-Fontcuberta et al. (17)	Cross-sectional	• 17 RIS • 17 CIS (12 months from onset) • 17 HCs	3 T	DTI	Voxel-based analysis of FA and MD maps in WM	• **FA in NAWM** • RIS = HCs • **Cerebellar WM** • CIS < HCs = RIS	NA
Labiano-Fontcuberta et al. (20)	Cross-sectional	• 18 RIS • 18 HCs	3 T	• ^1^H-MRSI • DTI	• Single voxel mid-parietal • GM: • NAA, Cr, Cho, MI, and Glx (absolute and ratios) • Voxel-based analysis of FA and MD maps in WM	No differences between groups	No differences between groups^a^
Mato-Abad et al. (21)	Cross-sectional	• 17 RIS • 17 CIS (at the onset)	3T	• 3D T1 • DTI	• FreeSurfer (Talairach atlas) • Voxel-based analysis of FA and MD maps in NAWM	L rostral middle frontal gyrus volume + FA R amygdala and lingual gyrus discriminating between RIS and CIS with 78% accuracy	NA
**Spinal cord**							
Zeydan et al. (22)	Cross-sectional	• 34 RIS • 31 RRMS • 25 SPMS (12 months from progression)	3T	T2 axial	• C2 area • C7 area • Average area between C2 and C7 (CASA)	• C2, C7 and CASA • RIS = RRMS > SPMS	NA
Alcaide-Leon et al. (23)	Cross-sectional	• 24 RIS • 14HCs	3T	• PSIR • DTI • MT	• Average area between C2 and C4 • FA, MD • MTr	No differences between groups	NA
**Central vein sign**							
Suthiphosuwan et al. (24)	Cross-sectional	20 RIS	3T	• 3D T1 • 3D T2-FLAIR • 3D T2* EPI	NA	90% RIS had ≥40% CVS +ve WMLs	NA
Oh et al. (25)	Cross-sectional	27 RIS	3T	• 3D T1 • 3D T2-FLAIR • 3D T2* EPI	NA	93% RIS had ≥40% CVS +ve WMLs	NA
George et al. (26)	Cross-sectional	5 RIS	7T	• 3D T1 • 3D T2-FLAIR • 3D T2* EPI	NA	All RIS had majority CVS +ve WMLs	NA
**Connectivity**							
Giorgio et al. (27)	Cross-sectional	• 18 RIS • 20 RRMS • 20 HCs	1.5T	• T1 • PD/T2 • DTI • rs-fMRI	• FSL • Voxel-based analysis of FA, AD and RD maps	• Altered WM tract integrity in RIS, RRMS • Altered FC in RRMS	NA
**Retina and optic nerve**							
Filippatou et al. (28)	Cross-sectional	• 30 RIS • 60 HC	NA	OCT	NA	• **GCIPL** • RIS with SC/IT lesions < RIS without SC/IT lesions, HC	NA
Knier et al. (29)	Longitudinal (1 year FU)	• 20 RIS • 18 CIS • 18 NSWML • 19 HC	3T	• OCT • T1 gradient echo • T2-FLAIR	NA	• **mRNFL** • CIS = RIS < HC = NSWML • **GCIPL** • RIS < CIS < HC = NSWML • **INL** • RIS = CIS > HC	• mRNFL • new lesions < stable RIS • INL • new lesions > stable RIS
Vural et al. (18)	Longitudinal (5 years median FU)	• 15 RIS • 15 HCs	NA	OCT	NA	• **GCIPL, mRNFL, pRNFL** • RIS < HCs	• pRNFL • MS converter < RIS
Aly et al. (30)	Longitudinal (6 years median FU)	• 36 RIS • 36 HCS	NA	OCT	NA	• **GCIPL, pRNFL** • RIS < HCs • **INL** • RIS = HCs	• GCIPL • pRNFL (baseline and over time) • MS converter < RIS

a Presence/absence of spinal cord lesions + ≥2 of the following characteristics: CSF abnormalities, gadolinium-enhancing lesions, dissemination in time of brain lesions.

b Presence/absence of CSF abnormalities, spinal cord lesions, or dissemination in time of brain lesions.

AD, axial diffusivity; CASA, cervical spinal cord average cross-sectional area; CIS, clinically isolated syndrome; Cho, choline; Cr, creatinine; CVS, central vein sign; DTI, diffusion tensor imaging; EPI, echo-planar imaging; FA, fractional anisotropy; FC, functional connectivity; FLAIR, fluid-attenuated inversion recovery; FU, follow-up; GCIPL, ganglion cell-inner plexiform layer; GLx, glutamine-glutamate complex; GM, grey matter; HCs, healthy controls; INL, inner nuclear layer; MD, mean diffusivity; MI, myoinositol; MT, magnetisation transfer; MTr, magnetisation transfer ratio; NAA, N-acetyl aspartate; NAWM, normal-appearing white matter; NSWML, non-specific white matter lesions; OCT, optical coherence tomography; pRNFL, peripapillary retinal nerve fibre layer; PD, proton density image; PSIR, phase-sensitive inversion recovery; R, right; RD, radial diffusivity; RIS, radiologically isolated syndrome; RRMS, relapsing-remitting multiple sclerosis; rs-fMRI, resting-state functional MRI; SPMS, secondary-progressive multiple sclerosis; WM, white matter; WML, white-matter lesions; ^1^H-MRSI, magnetic resonance spectroscopic imaging.

Labiano-Fontcuberta et al. (17) compared RIS with CIS at 12 months after onset with 3T MRI. They reported no differences in brain T2-white matter (WM) lesion load, but T2-lesion load correlated with cortical volume in both groups. Whilst participants with RIS had lower cortical and thalamic volumes than controls, those with CIS did not. Specific cortical thinning patterns also emerged in the RIS population but did not survive Bonferroni correction for multiple comparisons. Although WM volumes were similar between groups, voxel-based analysis using diffusion-weighted imaging (DWI) showed lower fractional anisotropy (FA; i.e., a marker of WM fibre integrity) (31) in the cerebellar WM in the CIS population compared with healthy controls.

The same group also characterised differences between RIS and healthy controls using ^1^H-MRSI and diffusion tensor imaging (DTI)-derived FA and mean diffusivity (MD) maps with 3T MRI (20). People with RIS showed lower brain and cortical volumes than controls, but contrary to previous findings by Stromillo et al. (11), they did not show alterations in NAA/Cho and Cr/Cho nor in white matter FA and MD.

A recent study (21) applied a machine-learning approach to differentiate RIS from CIS using multi-modal 3T MRI, which included cortical thickness, cortical and subcortical GM volumes, and DTI measures of WM integrity. A model of three features (left rostral middle frontal gyrus volume and FA of the right amygdala and lingual gyrus) could discriminate between RIS and CIS with an accuracy of 78%.

Interestingly, another recent study (19) exploring brain volumetrics at 3T in RIS reported a decrease in cerebellar WM and anterior GM compared with healthy controls. No differences were observed in total brain volume and cortical or deep GM volume between RIS and healthy controls.

### 2.2. Spinal cord MRI

Spinal cord lesions in RIS increase the risk of conversion to MS (2). In total, two studies have also investigated spinal cord atrophy and quantitative spinal cord MRI alterations in RIS.

Zeydan et al. (22) did not find differences in spinal cord average cross-sectional area at C2 and C7 between RIS and RRMS even though people with RRMS had more frequent cervical cord lesions (91%) than those with RIS (44%). Cervical spinal cord atrophy was only present in people with secondary progressive MS.

Alcaide-Leon et al. (23) assessed spinal cord DTI and MT metrics in RIS vs. healthy controls. The spinal cord cross-sectional area was measured between C3 and C4 and reported decreased brain volume in RIS compared with controls, with no evidence of cervical spinal cord atrophy. Furthermore, quantitative MRI analysis revealed weak evidence for lower spinal cord MT ratio in RIS compared with healthy controls.

### 2.3. Central vein sign

Iron-sensitive MRI sequences (such as T2^*^- and susceptibility-weighted imaging) can identify veins within demyelinating white-matter (WM) lesions, called the “central vein sign” (CVS) (32). The proportion of WM lesions that display the CVS can help distinguish between MS and other conditions associated with white-matter disease. A cutoff threshold of 40% of WM lesions displaying the CVS sign has been proposed (32)—proportions above this would be consistent with MS. An alternative proposal suggested a minimum of six lesions showing the CVS to support MS (33).

In total, three studies have considered the CVS in RIS. Suthiphosuwan et al. (24) analysed the number of WM lesions displaying the CVS in RIS and compared this with the reported proportion of CVS-positive WM lesions in RRMS. Out of 20 participants recruited, 18 (90%) had at least 40% of WM lesions showing the CVS and 19 (95%) had at least six lesions with the CVS. The two people who did not meet the 40% threshold had a relative paucity of imaging features consistent with RIS—for example, most lesions were small and punctate and were located in the anterior subcortical and deep WM. Regardless, these two participants had 29 and 31% of lesions demonstrating the CVS.

The same group also assessed the association of the CVS with cognitive impairment (25). In their cohort of 27 people with RIS, 25 (97%) had at least 40% of WM lesions displaying the CVS. The proportion of CVS-positive WM lesions predicted performance on the California Verbal Learning Test, an assessment of verbal memory.

The third study (26) performed a retrospective analysis of people diagnosed with RIS at their centre. Most of the 89 people with RIS did not undergo imaging sequences that would identify the CVS. Of the five that had T2^*^-weighted imaging, all met the same 40% threshold of WM lesions with CVS.

### 2.4. Connectivity

Advanced MRI techniques can also be used to assess brain connectivity. This might be with multi-shell DWI to estimate trajectories of WM tracts and examine structural connectivity or with resting-state functional MRI to review functional connectivity between different brain regions (34). Graph theory can then be applied to interrogate the integrity of the whole brain network (35)—for example, a comparison of CIS brain networks and those derived from healthy controls identified differences in network organisation between the two groups (36).

Only one study to date has assessed brain connectivity in RIS. Giorgio et al. (27) compared connectivity between RIS, RRMS, and healthy controls. Their voxel-wise analysis identified no differences between people with RIS and RRMS, though differences were noted with analysis of WM tract integrity. DTI metrics [FA, MD and radial diffusivity (RD)] were altered in RIS in regions with WM lesions but preserved in normal-appearing WM; they were altered in both WM lesions and normal-appearing WM in people with RRMS. Furthermore, a less conservative statistical analysis (significance *p* < 0.05, rather than < 0.01) showed lower axial diffusivity (AD) and RD in certain WM tracts incorporating both WM lesions and normal-appearing WM, such as the corpus callosum and corticospinal tract, in RIS compared with RRMS.

The same study (27) also examined functional connectivity using resting-state functional MRI. In total, two of the 12 resting-state networks had lower functional connectivity in RIS than RRMS: the sensorimotor network and right working memory network. However, there were no differences in these networks between RIS and HCs. There were no voxel-wise differences in brain functional connectivity between HCs and people with RIS.

### 2.5. Retinal layer imaging

Retinal layer thinning, as measured by optical coherence tomography (OCT), is considered a biomarker for neurodegeneration in MS (37). Some studies have assessed its role in RIS. Filippatou et al. (28) assessed OCT in RIS and healthy controls. Interestingly, no OCT differences were seen between RIS and healthy controls. However, there were associations between the presence of either spinal cord or infratentorial lesions and a reduced macular ganglion cell-inner plexiform layer (GCIPL). As spinal cord lesions increase the risk of conversion from RIS to MS (2), GCIPL thinning may similarly indicate high risk, though there was no follow-up to assess this. No associations were seen between OCT metrics and other risk factors for conversion to MS, such as CSF-unique oligoclonal bands or MRI-enhancing lesions.

Knier et al. (29) analysed the association of retinal thicknesses with inflammatory activity in RIS and CIS. They noted macular retinal nerve fibre layer (mRNFL) thinning and inner nuclear layer (INL) thickening in RIS and CIS compared with healthy controls. GCIPL was the lowest in the RIS group, though it was lower in CIS than HC. When MRI was repeated after 1 year, the appearance of new inflammatory lesions correlated with a thinner baseline GCIPL and a thicker baseline INL. No clinical activity was reported in the RIS group—the MRI changes merely represented DIT rather than conversion to MS; however, DIT is also a risk factor for conversion from RIS to MS (1).

Overall, two more longitudinal studies have been reported. Vural et al. (18) found reduced brain and thalamic volumes in RIS compared with healthy controls. OCT analysis revealed thinner GCIPL, mRNFL, and temporal peripapillary RNFL (pRNFL) in RIS. In RIS, these retinal metrics correlated with brain and thalamic volumes. Participants with RIS were followed-up for a median of 5 years: four out of 15 participants experienced a clinical episode, converting to MS. The pRNFL was thinner in individuals with RIS converting to MS than in those who did not convert. The groups were otherwise similar regarding T2-lesion load, with a presence or absence of gadolinium-enhancing lesions and volumetric measurements at the baseline.

Another longitudinal study (30) followed up participants with RIS for 6 years. People with RIS had thinner pRNFL and GCIPL than healthy controls. A total of eight out of 36 individuals converted to MS at follow-up. Conversion to MS was associated with thinning of the pRNFL and GCIPL at baseline and over time. After adjusting for other factors (age, sex, immunotherapy, and the occurrence of spinal cord lesions), Cox proportional hazards regression revealed a hazard ratio of 1.08 for conversion to MS for each 1 μm decline in pRNFL.

**Figure 1:**
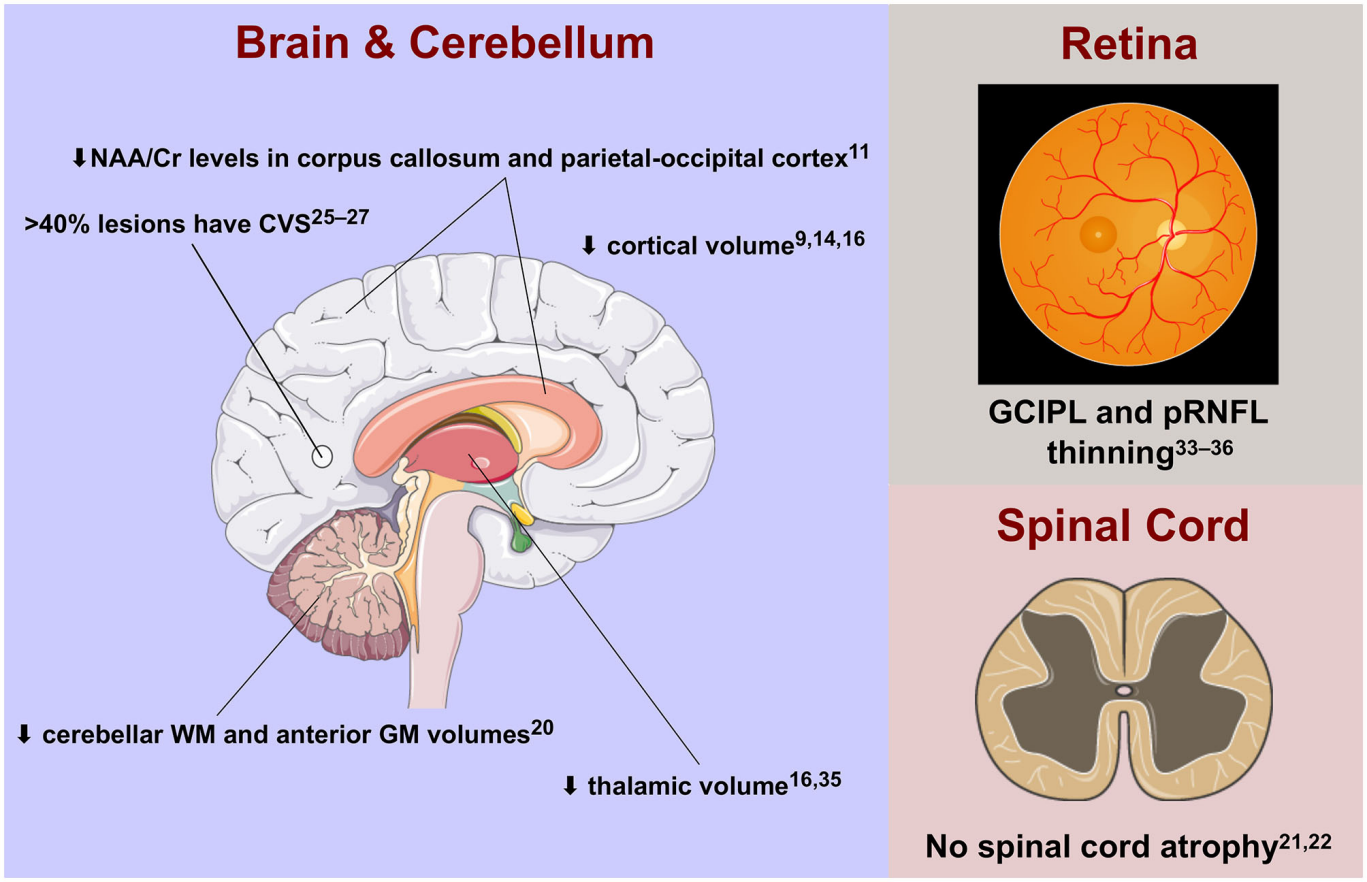
Advanced imaging features of radiologically isolated syndrome in the central nervous system. Cr, creatinine; CVS, central vein sign; GCIPL, ganglion cell-inner plexiform layer; GM, grey matter; NAA, N-acetyl aspartate; pRNFL, peripapillary retinal nerve fibre layer; WM, white matter.

Figure 1 summarises the mini-review findings.

## 3. Discussion

Most studies (9, 14, 15, 17, 18) have demonstrated a reduction in brain volumes, mainly GM, in RIS. Early studies showed global brain and GM atrophy. Later studies (17, 18) highlighted the contribution of deep GM structures to the observed changes in total GM volumes, particularly the thalamus. Thalamic atrophy can be present in early RRMS (16), including CIS (38) and paediatric onset MS (39), and it is related to disability (40) and fatigue (41). The thalamus could therefore be an early site of neurodegeneration in RIS. Longitudinal studies are required to confirm if thalamic involvement is associated with MS conversion.

The studies of volumetric brain MRI in RIS show some discrepancies. Whilst two studies (9, 14) observed generalised atrophy, even comparable to patients already diagnosed with RRMS (9), others (15, 17, 19) did not replicate these findings and found only regional volume loss. A possible reason relates to the small cohort sizes, making results difficult to generalise. RIS is undoubtedly a rare entity, and there is a lack of large-cohort studies. In addition, the cohorts recruited by the various studies differ in their clinical and radiological characteristics. De Stefano et al. (9), for instance, studied a cohort of RIS characterised by a high lesion volume (6.7 ± 6.5 cm^3^) similar to the control RRMS group, with cognitive deficits in 6/16 patients and DIT in 10/19 patients. These clinical and radiological data could explain why these individuals with RIS presented with brain atrophy like people with RRMS. Other studies have included cohorts of individuals with lower lesion burden [i.e., George et al. (19) 3.8 ± 2.9 cm^3^] or did not account for DIT or cognitive deficits (15). The presence of DIT and clinical signs may indeed suggest a longer duration of RIS that could lead to significant volumetric changes in the brain. Only longitudinal studies of larger cohorts will elucidate whether the presence of marked brain atrophy is a strong risk factor for conversion to RRMS.

Interestingly, spinal cord atrophy, a clinically relevant feature of MS (42), seemed not to be present in RIS, even though asymptomatic cord lesions are one of the main risk factors for MS conversion in RIS (2). These findings (22, 23) suggest that neurodegeneration in this area may manifest at a more advanced clinical stage. Further studies—possibly using new registration-based methods (43) or quantitative MRI protocols on the whole neuroaxis (44) in longitudinal cohorts—should shed more light on spinal cord involvement in RIS.

Instead, GCIPL and pRNFL atrophy, other biomarkers of neurodegeneration in MS (37), are present in RIS and are associated with risk factors for conversion to MS (28, 29) and with MS conversion itself (18, 30), Retinal atrophy is seen as early in the disease course as CIS (45, 46), and it is also related to clinical disability in MS (47). If confirmed, these results might suggest OCT as a tool to stratify disease severity, even as early as RIS.

The CVS also shows promise in RIS. Most individuals with RIS exceed the threshold of 40% of WM lesions demonstrating the sign (24–26), which can distinguish MS from other conditions with similar radiological appearances (32). Furthermore, a higher proportion of lesions displaying this sign correlates with deterioration in verbal working memory (25), However, if more than 90% of people with RIS meet the 40% CVS threshold (as reported in the identified literature), its utility in predicting conversion to MS may be limited—only longitudinal studies will be able to determine this. A different threshold may need to be established for the purposes of RIS risk stratification.

Advanced MRI imaging has helped to study the histopathological mechanisms of MS *in vivo* (13). As reported in this review, most of the studies conducted so far have not found convincing microscopic alterations in the brain and spinal cord of people with RIS. Conversely, quantitative MRI in early MS seems instead to reveal alterations not visible with conventional MRI (48). Therefore, one could hypothesise that alterations in normal-appearing tissues appear when the disease becomes clinically evident. However, the lack of longitudinal quantitative studies makes it difficult to draw firm conclusions.

Only one study (11) using ^1^H-MRSI revealed metabolic alterations in the cortex, lesional WM, and normal-appearing WM of RIS. Authors found a marked decrease in the NAA/Cr ratio (~10%), which is higher than that observed in some MS studies (49). However, this alteration was not related to lesion load, brain atrophy, or prognostic markers for MS conversion. Therefore, its clinical significance requires further exploration.

The role of connectivity analysis in RIS is still unclear—only one study has been published, which did not include longitudinal follow-up (27). The results do support the theory above—that alterations in the normal-appearing tissue appear when the disease is clinically evident—with the differences in WM tract integrity between RIS and MS: DTI metrics were altered in RIS only in areas with inflammatory lesions, whereas alterations were seen in MS in both lesional and normal-appearing WM. The same study's analysis of functional connectivity suggests that connectivity is stronger in two brain networks in RRMS compared with RIS and healthy controls—the increase in connectivity in RRMS might indeed represent maladaptive reorganisation, which is not yet required in the subclinical RIS. However, since this study, more novel techniques for generating and analysing connectivity maps have been developed—future analyses could incorporate these in a longitudinal setting.

There may be an additional role for machine learning—Mato-Abad et al. (21) were able to discriminate between people with RIS and CIS when synthesising the results of different imaging modalities. However, their sample size was small, and they did not report the results of any model performance testing: it is possible that the model is over-fitted to the tested cohort. It has not yet been applied to an independent dataset.

Overall, our understanding of RIS using these techniques is limited by the generally small sample sizes analysed. Indeed, some of the studies reviewed here are derived from analyses on common datasets: more than half of the studies into quantitative MRI in RIS are from the same patient cohort, for instance (17, 20, 21); a similar proportion of the central vein sign studies are also drawn from a common population (24, 25). Furthermore, few studies have had findings tested in independent datasets, and opposing findings were reported in studies assessing brain volumetrics. Considering the finding that dimethyl fumarate can delay the conversion of RIS to multiple sclerosis (5), it will become increasingly important to identify those at greatest risk of clinical disease. Larger collaborative datasets will therefore be required to progress research into advanced imaging techniques in RIS.

## 4. Conclusion

Although advances in CNS imaging have significantly improved our understanding of MS, there is a relative paucity of imaging studies in RIS. Whilst having homogeneous cohorts of RIS across centres may be challenging, the enrolment of larger cohorts in longitudinal studies could improve our understanding of this syndrome.

## Author contributions

MF and SC performed literature review and wrote the first draft of the manuscript. AT developed the topic for the review. All authors contributed to manuscript revision, read, and approved the submitted version.

## References

[1] Okuda DT Mowry EM Beheshtian A Waubant E Baranzini SE Goodin DS. Incidental MRI anomalies suggestive of multiple sclerosis: the radiologically isolated syndrome. Neurology. (2009) 72:800–5. 10.1212/01.wnl.0000335764.14513.1a 19073949

[2] Okuda DT Mowry EM Cree BAC Crabtree EC Goodin DS Waubant E. Asymptomatic spinal cord lesions predict disease progression in radiologically isolated syndrome. Neurology. (2011) 76:686–92. 10.1212/WNL.0b013e31820d8b1d 21270417 PMC3053327

[3] Lebrun-Frenay C Kantarci O Siva A Sormani MP Pelletier D Okuda DT. Radiologically isolated syndrome: 10-year risk estimate of a clinical event. Ann Neurol. (2020) 88:407–17. 10.1002/ana.25799 32500558

[4] Thompson AJ Banwell BL Barkhof F Carroll WM Coetzee T Comi G. Diagnosis of multiple sclerosis: 2017 revisions of the McDonald criteria. Lancet Neurol. (2018) 17:162–73. 10.1016/S1474-4422(17)30470-2 29275977

[5] Okuda DT Kantarci O Lebrun-Frénay C Sormani MP Azevedo CJ Bovis F. Dimethyl fumarate delays multiple sclerosis in radiologically isolated syndrome. Ann Neurol. (2023) 93:604–14. 10.1002/ana.26555 36401339

[6] Hauser SL Oksenberg JR Lincoln R Garovoy J Beck RW Cole SR. Interaction between HLA-DR2 and abnormal brain MRI in optic neuritis and early MS. Neurology. (2000) 54:1859–61. 10.1212/WNL.54.9.1859 10802800

[7] Tintoré M Rovira A Brieva L Grivé E Jardí R Borrás C. Isolated demyelinating syndromes: comparison of CSF oligoclonal bands and different MR imaging criteria to predict conversion to CDMS. Mult Scler. (2001) 7:359–63. 10.1191/135245801701567069 11795456

[8] Lebrun C Bensa C Debouverie M Wiertlevski S Brassat D de Seze J. Association between clinical conversion to multiple sclerosis in radiologically isolated syndrome and magnetic resonance imaging, cerebrospinal fluid, and visual evoked potential: follow-up of 70 patients. Arch Neurol. (2009) 66:841–6. 10.1001/archneurol.2009.119 19597085

[9] De Stefano N Stromillo ML Rossi F Battaglini M Giorgio A Portaccio E. Improving the characterization of radiologically isolated syndrome suggestive of multiple sclerosis. PLoS ONE. (2011) 6:e19452. 10.1371/journal.pone.0019452 21559385 PMC3084867

[10] Grossman RI Gomori JM Ramer KN Lexa FJ Schnall MD. Magnetization transfer: theory and clinical applications in neuroradiology. Radiographics. (1994) 14:279–90. 10.1148/radiographics.14.2.8190954 8190954

[11] Stromillo ML Giorgio A Rossi F Battaglini M Hakiki B Malentacchi G. Brain metabolic changes suggestive of axonal damage in radiologically isolated syndrome. Neurology. (2013) 80:2090–4. 10.1212/WNL.0b013e318295d707 23635962

[12] De Stefano N Filippi M Miller D Pouwels PJ Rovira A Gass A. Guidelines for using proton MR spectroscopy in multicenter clinical MS studies. Neurology. (2007) 69:1942–52. 10.1212/01.wnl.0000291557.62706.d3 17998486

[13] Cortese R Collorone S Ciccarelli O Toosy AT. Advances in brain imaging in multiple sclerosis. Ther Adv Neurol Disord. (2019) 12:1756286419859722. 10.1177/1756286419859722 31275430 PMC6598314

[14] Rojas JI Patrucco L Míguez J Besada C Cristiano E. Brain atrophy in radiologically isolated syndromes. J Neuroimaging. (2015) 25:68–71. 10.1111/jon.12182 25307993

[15] Azevedo CJ Overton E Khadka S Buckley J Liu S Sampat M. Early CNS neurodegeneration in radiologically isolated syndrome. Neurol Neuroimmunol Neuroinflamm. (2015) 2:e102. 10.1212/NXI.0000000000000102 25884012 PMC4396526

[16] Azevedo CJ Cen SY Khadka S Liu S Kornak J Shi Y. Thalamic atrophy in multiple sclerosis: a magnetic resonance imaging marker of neurodegeneration throughout disease. Ann Neurol. (2018) 83:223–34. 10.1002/ana.25150 29328531 PMC6317847

[17] Labiano-Fontcuberta A Mato-Abad V Álvarez-Linera J Hernández-Tamames JA Martínez-Ginés ML Aladro Y. Gray matter involvement in radiologically isolated syndrome. Medicine. (2016) 95:e3208. 10.1097/MD.0000000000003208 27043685 PMC4998546

[18] Vural A Okar S Kurne A Sayat-Gürel G Acar NP Karabulut E. Retinal degeneration is associated with brain volume reduction and prognosis in radiologically isolated syndrome. Mult Scler. (2020) 26:38–47. 10.1177/1352458518817987 30526302

[19] George IC El Mendili MM Inglese M Azevedo CJ Kantarci O Lebrun C. Cerebellar volume loss in radiologically isolated syndrome. Mult Scler. (2021) 27:130–3. 10.1177/1352458519887346 31680617 PMC7196519

[20] Labiano-Fontcuberta A Mato-Abad V Álvarez-Linera J Hernández-Tamames JA Martínez-Ginés ML Aladro Y. Normal-appearing brain tissue analysis in radiologically isolated syndrome using 3 T MRI. Medicine. (2016) 95:e4101. 10.1097/MD.0000000000004101 27399108 PMC5058837

[21] Mato-Abad V Labiano-Fontcuberta A Rodríguez-Yáñez S García-Vázquez R Munteanu CR Andrade-Garda J. Classification of radiologically isolated syndrome and clinically isolated syndrome with machine-learning techniques. Eur J Meurol. (2019) 26:1000–5. 10.1111/ene.13923 30714276

[22] Taheri K Vavasour IM Abel S Lee LE Johnson P Ristow S. Cervical spinal cord atrophy. Neurol Neuroimmunol Neuroinflamm. (2018) 5:435. 10.1212/NXI.0000000000000435 29435472 PMC5795903

[23] Alcaide-Leon P Cybulsky K Sankar S Casserly C Leung G Hohol M. Quantitative spinal cord MRI in radiologically isolated syndrome. Neurol Neuroimmunol Neuroinflamm. (2018) 5:436. 10.1212/NXI.0000000000000436 29359174 PMC5773843

[24] Suthiphosuwan S Sati P Guenette M Montalban X Reich DS Bharatha A. The central vein sign in radiologically isolated syndrome. Am J Neuroradiol. (2019) 40:776–83. 10.3174/ajnr.A6045 31000526 PMC6786901

[25] Oh J Suthiphosuwan S Sati P Absinta M Dewey B Guenette M. Cognitive impairment, the central vein sign, and paramagnetic rim lesions in RIS. Mult Scler. (2021) 27:2199–208. 10.1177/13524585211002097 33754887 PMC8458475

[26] George IC Rice DR Chibnik LB Mateen FJ. Radiologically isolated syndrome: a single-center, retrospective cohort study. Mult Scler Relat Disord. (2021) 55:103183. 10.1016/j.msard.2021.103183 34365315

[27] Giorgio A Stromillo ML De Leucio A Rossi F Brandes I Hakiki B. Appraisal of brain connectivity in radiologically isolated syndrome by modeling imaging measures. J Neurosci. (2015) 35:550–8. 10.1523/JNEUROSCI.2557-14.2015 25589750 PMC6605365

[28] Filippatou A Shoemaker T Esch M Qutab M Gonzalez-Caldito N Prince JL. Spinal cord and infratentorial lesions in radiologically isolated syndrome are associated with decreased retinal ganglion cell/inner plexiform layer thickness. Mult Scler. (2019) 25:1878–87. 10.1177/1352458518815597 30507269 PMC6546560

[29] Knier B Berthele A Buck D Schmidt P Zimmer C Mühlau M. Optical coherence tomography indicates disease activity prior to clinical onset of central nervous system demyelination. Mult Scler. (2016) 22:893–900. 10.1177/1352458515604496 26362905

[30] Aly L Havla J Lepennetier G Andlauer TFM Sie C Strauß E-M. Inner retinal layer thinning in radiologically isolated syndrome predicts conversion to multiple sclerosis. Eur J Neurol. (2020) 27:2217–24. 10.1111/ene.14416 32589804

[31] Pierpaoli C Barnett A Pajevic S Chen R Penix LR Virta A. Water diffusion changes in wallerian degeneration and their dependence on white matter architecture. Neuroimage. (2001) 13:1174–85. 10.1006/nimg.2001.0765 11352623

[32] Preziosa P Rocca MA Filippi M. Central vein sign and iron rim in multiple sclerosis: ready for clinical use? Curr Opin Neurol. (2021) 34:505–13. 10.1097/WCO.0000000000000946 33928930

[33] Mistry N Abdel-Fahim R Samaraweera A Mougin O Tallantyre E Tench C. Imaging central veins in brain lesions with 3-T T2^*^-weighted magnetic resonance imaging differentiates multiple sclerosis from microangiopathic brain lesions. Mult Scler. (2016) 22:1289–96. 10.1177/1352458515616700 26658816

[34] Fornito A Zalesky A Breakspear M. Graph analysis of the human connectome: promise, progress, and pitfalls. Neuroimage. (2013) 80:426–44. 10.1016/j.neuroimage.2013.04.087 23643999

[35] Rubinov M Sporns O. Complex network measures of brain connectivity: uses and interpretations. Neuroimage. (2010) 52:1059–69. 10.1016/j.neuroimage.2009.10.003 19819337

[36] Tur C Grussu F Prados F Charalambous T Collorone S Kanber B. A multi-shell multi-tissue diffusion study of brain connectivity in early multiple sclerosis. Mult Scler. (2020) 26:774–85. 10.1177/1352458519845105 31074686 PMC7611366

[37] Graves JS Oertel FC Van der Walt A Collorone S Sotirchos ES Pihl-Jensen G. Leveraging visual outcome measures to advance therapy development in neuroimmunologic disorders. Neurol Neuroimmunol Neuroinflamm. (2021) 9:e1126. 10.1212/NXI.0000000000001126 34955459 PMC8711076

[38] Ontaneda D Raza PC Mahajan KR Arnold DL Dwyer MG Gauthier SA. Deep grey matter injury in multiple sclerosis: a NAIMS consensus statement. Brain. (2021) 144:1974–84. 10.1093/brain/awab132 33757115 PMC8370433

[39] Aubert-Broche B Fonov V Ghassemi R Narayanan S Arnold DL Banwell B. Regional brain atrophy in children with multiple sclerosis. Neuroimage. (2011) 58:409–15. 10.1016/j.neuroimage.2011.03.025 21414412

[40] Eshaghi A Prados F Brownlee WJ Altmann DR Tur C Cardoso MJ. Deep gray matter volume loss drives disability worsening in multiple sclerosis. Ann Neurol. (2018) 83:210–22. 10.1002/ana.25145 29331092 PMC5838522

[41] Capone F Collorone S Cortese R Di Lazzaro V Moccia M. Fatigue in multiple sclerosis: the role of thalamus. Mult Scler. (2020) 26:6–16. 10.1177/1352458519851247 31138052

[42] Moccia M Ruggieri S Ianniello A Toosy A Pozzilli C Ciccarelli O. Advances in spinal cord imaging in multiple sclerosis. Ther Adv Neurol Disord. (2019) 12:175628641984059. 10.1177/1756286419840593 31040881 PMC6477770

[43] Moccia M Prados F Filippi M Rocca MA Valsasina P Brownlee WJ. Longitudinal spinal cord atrophy in multiple sclerosis using the generalized boundary shift integral. Ann Neurol. (2019) 86:704–13. 10.1002/ana.25571 31385358

[44] Collorone S Cawley N Grussu F Prados F Tona F Calvi A. Reduced neurite density in the brain and cervical spinal cord in relapsing-remitting multiple sclerosis: a NODDI study. Mult Scler. (2020) 26:1647–57. 10.1177/1352458519885107 31682198

[45] Collorone S Kanber B Hashem L Cawley N Prados F Davagnanam I. Visual function and brief cognitive assessment for multiple sclerosis in optic neuritis clinically isolated syndrome patients. J Neuroophthalmol. (2022) 42:E22–31. 10.1097/WNO.0000000000001280 34561401 PMC8834161

[46] Pisa M Croese T Costa GD Guerrieri S Huang S-C Finardi A. Subclinical anterior optic pathway involvement in early multiple sclerosis and clinically isolated syndromes. Brain. (2021) 144:848–62. 10.1093/brain/awaa458 33829250

[47] Martinez-Lapiscina EH Arnow S Wilson JA Saidha S Preiningerova JL Oberwahrenbrock T. Retinal thickness measured with optical coherence tomography and risk of disability worsening in multiple sclerosis: a cohort study. Lancet Neurol. (2016) 15:574–84. 10.1016/S1474-4422(16)00068-5 27011339

[48] Collorone S Prados F Kanber B Cawley NM Tur C Grussu F. Brain microstructural and metabolic alterations detected *in vivo* at onset of the first demyelinating event. Brain. (2021) 144:1409–21. 10.1093/brain/awab043 33903905 PMC8219367

[49] Chard DT Griffin CM McLean MA Kapeller P Kapoor R Thompson AJ. Brain metabolite changes in cortical grey and normal-appearing white matter in clinically early relapsing-remitting multiple sclerosis. Brain. (2002) 125:2342–52. 10.1093/brain/awf240 12244090

